# TNFα signalling primes chromatin for NF-κB binding and induces rapid and widespread nucleosome repositioning

**DOI:** 10.1186/s13059-014-0536-6

**Published:** 2014-12-03

**Authors:** Sarah Diermeier, Petros Kolovos, Leonhard Heizinger, Uwe Schwartz, Theodore Georgomanolis, Anne Zirkel, Gero Wedemann, Frank Grosveld, Tobias A Knoch, Rainer Merkl, Peter R Cook, Gernot Längst, Argyris Papantonis

**Affiliations:** Department of Biochemistry III, University of Regensburg, Universität Strasse 31, 93053 Regensburg, Germany; Cell Biology and Genetics, Erasmus Medical Center, Wytemaweg 80, 3015 CN Rotterdam, The Netherlands; Institute of Biophysics and Physical Biochemistry, University of Regensburg, 93040 Regensburg, Germany; Centre for Molecular Medicine, University of Cologne, Robert-Koch-Strasse 21, 50931 Cologne, Germany; Institute for Applied Computer Science, University of Applied Sciences Stralsund, Zur Schwedenschanze 15, 18435 Stralsund, Germany; Biophysical Genomics, Erasmus Medical Center, Wytemaweg 80, 3015 CN Rotterdam, The Netherlands; BioQuant & German Cancer Research Center, Im Neuenheimer Feld 267, 69120 Heidelberg, Germany; Sir William Dunn School of Pathology, University of Oxford, South Parks Road, OX1 3RE Oxford, United Kingdom; Present address: Cold Spring Harbor Laboratory, 1 Bungtown Road, Cold Spring Harbor, 11724 NY USA

## Abstract

**Background:**

The rearrangement of nucleosomes along the DNA fiber profoundly affects gene expression, but little is known about how signalling reshapes the chromatin landscape, in three-dimensional space and over time, to allow establishment of new transcriptional programs.

**Results:**

Using micrococcal nuclease treatment and high-throughput sequencing, we map genome-wide changes in nucleosome positioning in primary human endothelial cells stimulated with tumour necrosis factor alpha (TNFα) - a proinflammatory cytokine that signals through nuclear factor kappa-B (NF-κB). Within 10 min, nucleosomes reposition at regions both proximal and distal to NF-κB binding sites, before the transcription factor quantitatively binds thereon. Similarly, in long TNFα-responsive genes, repositioning precedes transcription by pioneering elongating polymerases and appears to nucleate from intragenic enhancer clusters resembling super-enhancers. By 30 min, widespread repositioning throughout megabase pair-long chromosomal segments, with consequential effects on three-dimensional structure (detected using chromosome conformation capture), is seen.

**Conclusions:**

Whilst nucleosome repositioning is viewed as a local phenomenon, our results point to effects occurring over multiple scales. Here, we present data in support of a TNFα-induced priming mechanism, mostly independent of NF-κB binding and/or elongating RNA polymerases, leading to a plastic network of interactions that affects DNA accessibility over large domains.

**Electronic supplementary material:**

The online version of this article (doi:10.1186/s13059-014-0536-6) contains supplementary material, which is available to authorized users.

## Background

The arrangement of nucleosomes along the chromatin fibre profoundly affects genome function [1,2]. For example, silenced genomic segments and constitutive heterochromatin contain nucleosomes positioned in high-density arrays [1,3,4], whereas active and regulatory regions appear more disorganized and ‘open’ [1,5,6]. Although some data exist on the reorganization of the nucleosomal landscape following extra-cellular signalling [7,8] and differentiation [9,10], the temporally resolved dynamics of chromatin architecture remain poorly characterized.

Nucleosome positioning can be mapped genome-wide at single-nucleosome resolution using micrococcal nuclease digestion followed by sequencing (MNase-seq) [11,12]. We applied this technique to primary human umbilical vein endothelial cells (HUVECs) stimulated with tumour necrosis factor alpha (TNFα). This potent cytokine drives the inflammatory response by signalling through the transcription factor nuclear factor kappa-B (NF-κB) [13,14]; on phosphorylation, NF-κB translocates into nuclei, where it regulates hundreds of genes [15,16]. Therefore, we correlated nucleosomal repositioning with genome-wide NF-κB binding (assessed by chromatin immunoprecipitation coupled to high-throughput sequencing; ChIP-seq) and gene expression (assessed by sequencing of total RNA; RNA-seq).

**Figure 1:**
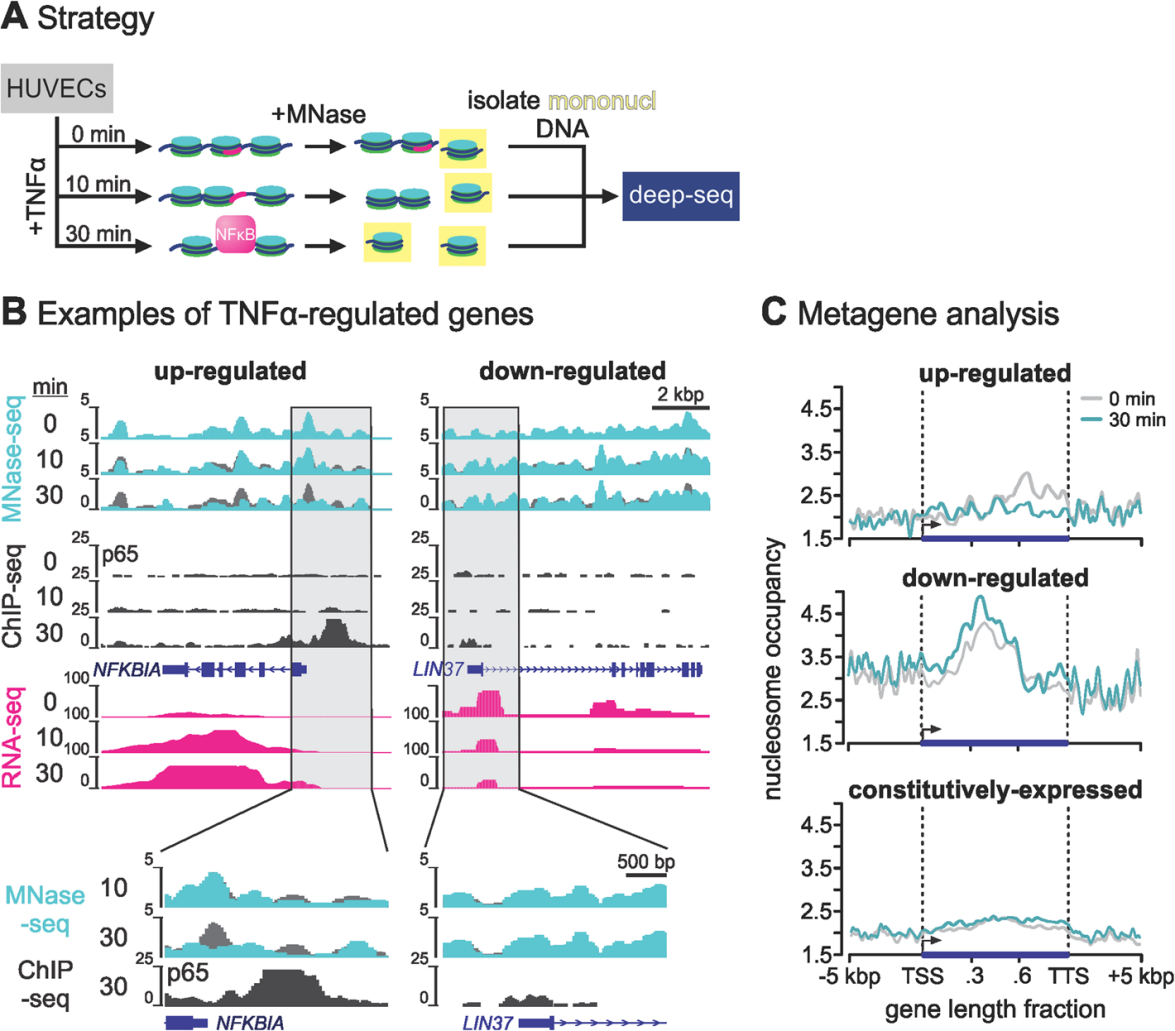
**Nucleosome repositioning in TNFα-responsive genes.** **(A)** Strategy: HUVECs were serum-starved and stimulated with ΤΝFα (0, 10, 30 min), treated with MNase, and DNA associated with mononucleosomes (*highlighted yellow*) deep-sequenced. Nucleosomes reposition within 10 min to unmask NF-κB binding sites (*magenta*), before NF-κB enters the nucleus. **(B)** Browser tracks (*vertical axes* - reads/million; magnifications of transcription start sites shown below) for typical up- or down-regulated genes obtained by MNase-seq (*green*; reflects nucleosomal profiles; 0-min levels in *grey* underlie 10- and 30-min ones to facilitate comparison), p65 ChIP-seq (*black*; reflects NF-κB binding), and total RNA-seq (*magenta*; reflects RNAPII activity). **(C)** Nucleosome occupancy (reads/million; MNase-seq) at 0 (*grey*) or 30 min post-stimulation (*green*) along metagenes derived from 109 up-regulated (>0.6 log_2_ fold-change at 30 compared to 0 min, plus >100 reads mapping to each), 69 down-regulated (<−0.6 log_2_ fold-change, plus >100 reads mapping to each), and 509 constitutively expressed genes (±0.01 log_2_ fold-change, plus >100 reads mapping to each). Genes were aligned at transcription start/termination sites (*dotted lines*), gene bodies divided into 50-bp windows, lengths scaled proportionately, and MNase-seq reads in each window summed; profiles from 5 kbp up- and downstream are also displayed. ChIP-seq, chromatin immunoprecipitation coupled to high-throughput sequencing; kbp, kilobase pair; MNase-seq, micrococcal nuclease digestion followed by sequencing; NF-κB, nuclear factor kappa-B; RNA-seq, sequencing of total RNA; TNFα, tumour necrosis factor alpha; TSS, transcription start site; TTS, transcription termination site.

We focused on spatial and temporal changes in chromatin architecture during the critical window when ‘immediately-early’ proinflammatory genes become active: 0, 10 and 30 min post-stimulation. In agreement with the idea that nucleosomes reposition in coincidence with (and/or as a result of) transcription factor binding at cognate sites [1–6], we did not expect to observe widespread repositioning before NF-κB binding was quantitatively detected (that is, 15 min post-stimulation [17,18]). However, we observed widespread nucleosome repositioning already by 10 min, coinciding with marginal, if any, stable binding of the factor (Figure 1A). Similarly, we expected elongation by pioneering RNA polymerases along TNFα-responsive genes to initiate a ‘wave’ of repositioning; however, examination of long (>100 kilobase pairs (kbp)) genes that are synchronously activated by TNFα showed that nucleosomes were already repositioned all the way from 5′ to 3′ ends, despite polymerases having transcribed <50% of their length after 30 min [19,20]. We attribute this to changes in positioning that nucleate from few selected NF-κB binding clusters embedded in the bodies of such responsive genes. We show that these effects are accompanied by changes in the three-dimensional conformation of the chromatin fibre - detected using chromosome conformation capture coupled to deep sequencing (3C-seq [21]).

## Results

### TNFα induces immediate widespread changes in nucleosome positioning

HUVECs grown to confluence were serum-starved (to promote synchrony), stimulated with TNFα for 0, 10 or 30 min, and treated with MNase to release mononucleosomes. The purified DNA (Additional file 1A) was deep-sequenced to obtain approximately 180 million read-pairs per time point (Figure 1A). When mapped to the reference genome (hg19), reads from two 0- and 30-min biological replicates gave comparable profiles (Additional file 1B).

First, we identified peaks in the MNase-seq read profiles that marked single-nucleosome positions (using findPeaks [22]) and selected those differentially unmasked at 10 or 30 min post-stimulation (that is, those where nucleosomes are repositioned by >10 bp when compared to 0 min). By 10 min, unmasked regions were enriched for binding motifs of proinflammatory transcription factors (for example, NF-κB, AP-1; Additional file 1C), and characterized by Gene Ontology terms associated with cell regulation and cytokine signalling (Additional file 1D). Notably, short interspersed nuclear elements [23], especially *AluY*, *AluSx* and *AluSg*, which all contain NF-κB binding sites [24] and confer enhancer-like characteristics [25], were amongst the most significantly unmasked regions (Table 1). These findings are perhaps surprising, because levels of nuclear NF-κB do not peak before 15 to 17.5 min (Additional file 1E) [18,26,27]. By 30 min, regulatory regions (for example, CpG islands, promoters, 5′ untranslated regions) and genes (for example, coding regions, exons) were all statistically significantly unmasked (Table 1). These data point to a progressive transition from the 0- to the 10-min, and finally to the 30-min, state.

**Table 1:** **Genome Ontology analysis of nucleosome-unmasked regions**

**10 versus 0 min TNFα stimulation**			**30 versus 0 min TNFα stimulation**		
**Annotation**	**GO group**	**log** ***P*** **-value**	**Annotation**	**GO group**	**log** ***P*** **-value**
rRNA	Basic	−132.6	CpG island	Basic	−18,894.5
-	-	-	coding	Basic	−2,508.6
-	-	-	protein-coding	Basic	−2,202.6
-	-	-	exons	Basic	−2,175.4
-	-	-	promoters	Basic	−1,637.2
-	-	-	5′ UTR	Basic	−1,573.7
-	-	-	rRNA	Basic	−90.7
-	-	-	miscRNA	Basic	−57.6
-	-	-	TTS	Basic	−30.3
-	-	-	miRNA	Basic	−2.3
Alu	SINE	−1,010,851.5	Alu	SINE	−127,523.7
Satellite	Satellite	−138,649.4	**AluY**	SINE	−254,77.1
**AluSx**	**SINE**	**−130,806.8**	AluJb	SINE	−13,157.4
Simple	Repeat	−109,102.9	**AluSx**	SINE	−10,162.6
AluSz	SINE	−95,618.8	**AluSx1**	SINE	−9,723.1
Satellite	Satellite	−84,407.6	AluSz	SINE	−7,150.9
TGn	Repeat	−82,907.0	AluJr	SINE	−6,259.1
Can	Repeat	−82,146.3	AluJo	SINE	−5,837.8
**AluSx1**	**SINE**	**−81,015.6**	AluSz6	SINE	−3,989.9
CATTCn	Satellite	−77,388.9	**AluSg**	SINE	−3,556.9

A list of the top regions unmasked at 10 and 30 min post-stimulation (looking at nucleosomes identified using findPeaks [23] that were repositioned by >10 bp at 10 or 30 compared to 0 min). *Top half*: regions associated with ‘basic’ genome annotation. *Bottom half*: repeat elements. For each entry, the annotation category, genome ontology group and identification confidence levels (log *P*-value) are shown; *Alu* repeats known to bind NF-κB [24] are in bold. GO, Gene Ontology; SINE, short interspersed nuclear elements; TNFα, tumour necrosis factor alpha.

### TNFα induces repositioning in differentially regulated gene subsets

We next examined genes differentially regulated following a 30-min TNFα pulse. They were selected using data obtained after deep sequencing total rRNA-depleted RNA (RNA-seq; approximately 120 million read pairs per time point) and were required to change by at least ±0.6 log_2_-fold (that is, ±1.5-fold at 30 min relative to 0 min); constitutively expressed genes (±0.01 log_2_-fold) provided controls (Additional file 2A and Additional file 3). We also monitored NF-κB binding using ChIP-seq data (by targeting its p65 subunit) at 10 and 30 min post-stimulation. At 10 min, marginal binding was observed, in agreement with data showing that NF-κB translocation into the nucleus and binding to cognate sites is not quantitatively detected before 15 or 30 min, respectively (examples in Figure 1B and Additional file 1E). At 30 min, more than 80% of up-regulated genes were associated with at least one p65 peak, compared to just 10% of down-regulated ones (compared to 6% and 7% for the 10-min data; Additional file 2B).

Comparison of MNase-seq (raw) read profiles along a typical immediate-early up-regulated gene, *NFKBIA*, showed nucleosomes already repositioned by 10 min, and changes in nucleosome occupancy became more pronounced at 30 min, when density decreased throughout the locus as NF-κB binding increased (Figure 1B, *left*). By contrast, profiles on a typical down-regulated gene, *LIN37*, became heightened and more defined (Figure 1B, *right*). This held true for other up- or down-regulated genes, whilst those of constitutively expressed loci varied little (Additional file 2C).

Global changes in genic nucleosome occupancy were assessed using ‘metagene’ analyses, by aggregating profiles from all up- or down-regulated genes. In up-regulated genes, the first few nucleosomes downstream of the promoter became more precisely positioned (most likely as transcription start site (TSS)-proximal nucleosomes form well-positioned arrays [1]), and occupancy decreased incrementally towards the 3′ end (as nucleosome-rich exons tend to be found more 3′ [28,29]). In down-regulated genes, occupancy increased throughout; again, little change was observed in constitutively expressed loci (Figure 1C).

### Nucleosome repositioning precedes transcriptional elongation in long genes

**Figure 2:**
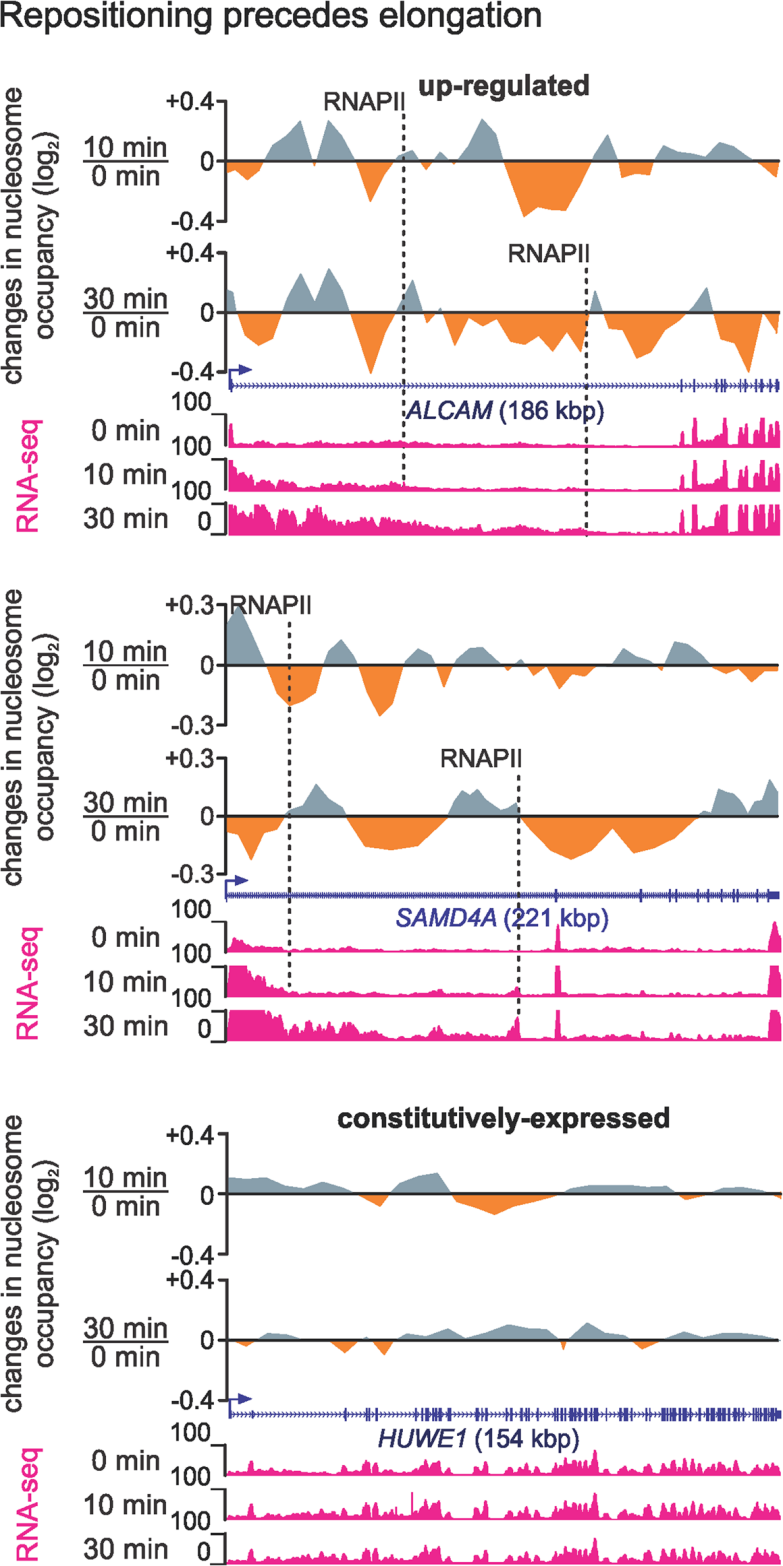
**Nucleosome repositioning at 3ʹ ends of long genes precedes transcription by pioneering (elongating) polymerases.** Browser views show (log_2_ fold) changes in nucleosome occupancy 10 or 30 min post-stimulation calculated using 5-kbp non-overlapping windows and a running-means average along up-regulated long genes *ALCAM* and *SAMD4A*. Changes (read enrichment - *grey*; read depletion - *orange*) are shown normalized to those in transcriptionally inert genomic regions. Total RNA-seq tracks (*magenta*) show elongating polymerases generating intronic signal close to the 5′ ends of genes after 10 and 30 min, as they have not yet reached termini (*dotted lines* - positions of pioneering RNAPs after 10 and 30 min). The long, constitutively expressed *HUWE1* locus (*bottom*) serves as a control. Kbp, kilobase pair; RNAP, RNA polymerase; RNA-seq, sequencing of total RNA.

The transcriptional activation of five long genes of >100 kbp has been studied in detail in this experimental model [17–20]. Following treatment with TNFα, pioneering RNA polymerases (RNAPs) initiate synchronously at the TSSs within 15 min, and then elongate at approximately 3 kbp/min. Thus, elongating RNAPs have transcribed less than the first half of these long genes after 30 min (see RNA-seq profiles in Figure 2 and ChIP-quantitative PCR (qPCR) in Additional file 4A). Therefore, one would expect nucleosomes only in the first half of these genes to have been repositioned.

To simplify analysis, we initially applied the PeakPredictor algorithm [30] to our MNase-seq data and ‘called’ single-nucleosome positions along three such long genes. As expected, TSS-proximal regions appeared progressively more depleted of nucleosome peaks (for example, in the first 10 kbp downstream of the TSS of 318-kbp *EXT1*, 41, 38 and 24 peaks were called at 0, 10 and 30 min, respectively; Additional file 4B). Unexpectedly, peak depletion of the same scale spread over hundreds of kilobase pairs from TSS to transcription termination site (TTS) (for example, the number of peaks throughout *EXT1* fell by 12% after 30 min; Additional file 4B), and ‘MNase-on-ChIP’ verified this effect (Additional file 4C).

Of course the above effect does not accurately describe the phenomenon, as there exist no such long nucleosome-devoid stretches of DNA. Thus, we analysed MNase-seq data throughout each long gene via a custom bioinformatics pipeline to examine whether nucleosome repositioning follows RNAP elongation (as might be expected). Genes were divided into 5-kbp non-overlapping windows, and changes in each window scored relative to (background) levels of nucleosome repositioning occurring in transcriptionally inert genomic segments (see Methods). This revealed a decrease in nucleosome occupancy (hereafter termed depletion), which was evident throughout 186-kbp *ALCAM* and 221-kbp *SAMD4A* (Figure 2), as well as in 116-kbp *NFKB1* and 458-kbp *ZFPM2* (Additional file 5A), at both 10 and 30 min, when pioneer RNAPs had advanced for <30 and <100 kbp, respectively. This effect was reproducible between biological replicates (Additional file 5B), and profiles of down-regulated and constitutively expressed genes served as controls (Figure 2 and Additional file 5A).

### NF-κΒ binding is associated with repositioning over great distances

**Figure 3:**
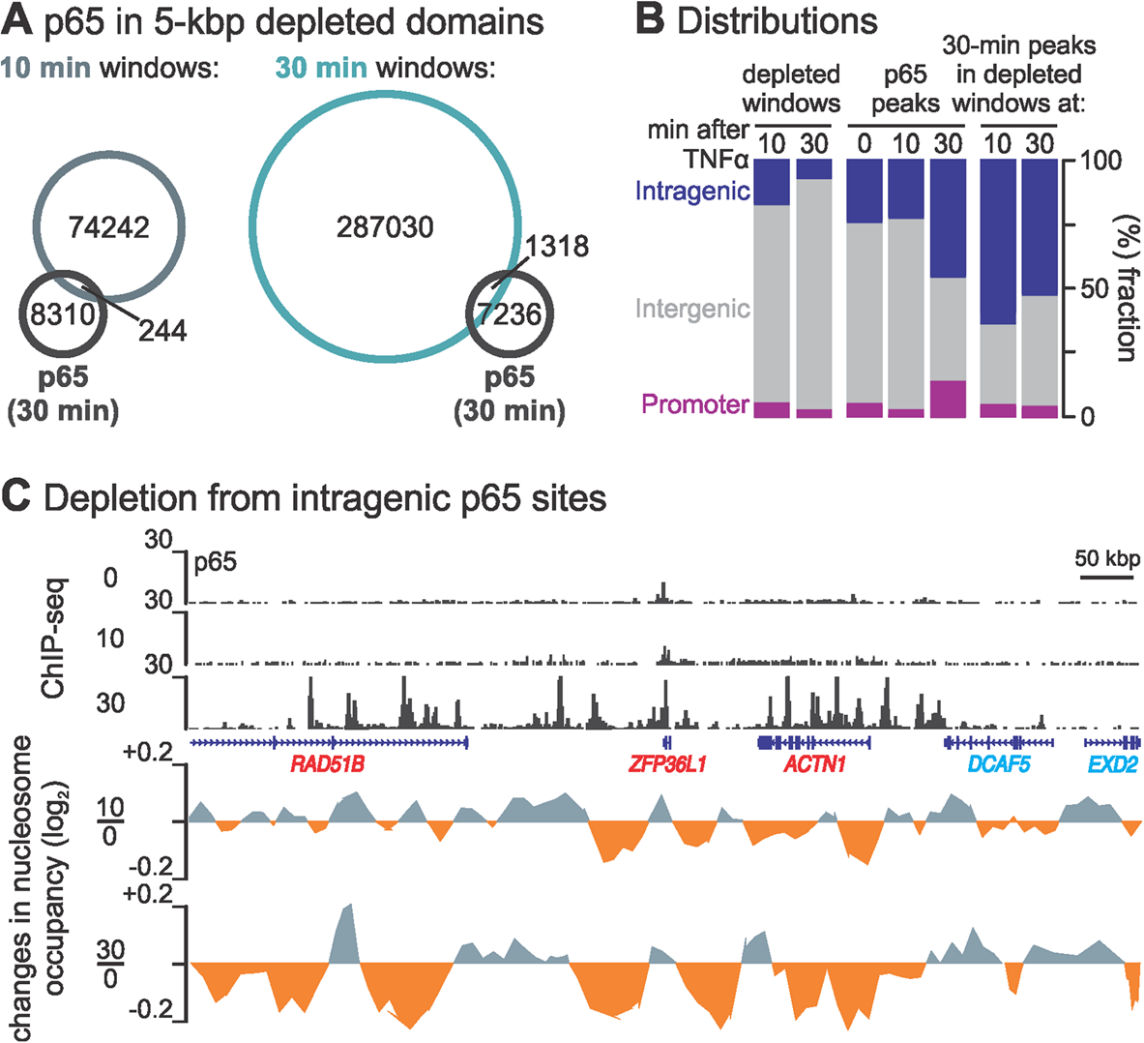
**NF-κB binding in TNFα-regulated nucleosomal domains. (** **A)** A minority of p65 peaks are found in depleted domains. The genome was partitioned into 5-kbp non-overlapping windows, and those depleted of nucleosomes selected (determined as in Figure 2) and compared to the location of p65 binding sites (determined using ChIP-seq data obtained 30 min post-stimulation). By 10 min, 74,486 nucleosome-depleted windows appear, after 30 min 288,377 such windows develop (21,788 of which are also seen at 10 min). By 30 min, 8,554 p65 peaks are seen, but only 244 and 1,318 overlap (≥25% of sequence) with the 10- and 30-min nucleosome-depleted windows, respectively. **(B)** Nucleosome-depleted p65-containing windows are predominantly intragenic. Bar graphs give the fraction of nucleosome-depleted windows or p65 peaks (0, 10, 30 min) coinciding with regions lying within or outside annotated genes (*blue* - intragenic; *grey* - intergenic), or ±1 kbp from the transcription start site (*purplr* - promoter). **(C)** Browser tracks illustrating changes in nucleosome occupancy (log_2_ fold-changes determined using 5-kbp non-overlapping windows as in Figure 2) in a 1-Mbp locus on chromosome 14 (TNFα-responsive genes - *red*, non-responsive - *blue*); p65 ChIP-seq tracks (0, 10 and 30 min post-TNFα; *vertical axes* - reads/million) are also shown. ChIP-seq, chromatin immunoprecipitation coupled to high-throughput sequencing; kbp, kilobase pair; TNFα, tumour necrosis factor alpha.

We next examined whether NF-κB binding was enriched in kilobase pair-long genomic segments displaying reduced MNase-seq signal. ChIP-seq collected 10 min post-stimulation showed sparse binding of p65 (approximately 200 peaks genome-wide, most at repeat elements; Additional file 6A), but by 30 min around 8,600 peaks were detected, most found at sites bearing histone marks characteristic of enhancers (high H3K4me1 and H3K27ac, low H3K4me3 [31]; Additional file 6A). At the same time, >280,000 5-kbp windows appeared depleted of nucleosomes (defined as above). Remarkably, <20% of p65 peaks (1,318) were embedded in such depleted windows, and the overlap was even smaller when compared to 10-min windows (244 peaks; Figure 3A). This is inconsistent with a simple model where NF-κB binding drives genome-wide nucleosome depletion, especially as little NF-κB has quantitatively bound in HUVEC chromatin by 10 min (Figure 1B and Additional file 1E). Intriguingly, p65-bearing windows significantly associated with gene bodies (Figure 3B).

As p65 binds both close to and in the body of many up-regulated genes (Additional file 2B), we speculated that the TNFα-driven repositioning seen throughout such genes (Figure 1C) might be nucleated from p65 bound at intragenic sites (Figure 3C illustrates one locus). Thus, of all up-regulated genes examined, 72% encompassed ≥1 p65 peak; by contrast, <10% of down-regulated genes contained a p65 peak (Additional file 7A). The physical separation between such intragenic peaks in up-regulated genes is an order of magnitude greater than those between intergenic ones (despite the small fraction of the genome occupied by protein-coding genes); thus, this group of peaks covers a substantial portion of the respective gene bodies (Additional file 7A). These results point to a focused binding of NF-κΒ, in clusters of ‘primed’ sites, within genes (even though the transcription factor might be bound at low titres), followed by nucleosome repositioning over several tens of kilobase pairs (Additional file 6B and Additional file 7B).

### Multi-scale nucleosome repositioning impacts on higher-order structure

We next used the long arm of chromosome 14 as a model to study how changes in nucleosome density might affect structure at increasingly larger scales (as loci on this chromosome have been extensively studied before [17–20]). The chromosome was divided into non-overlapping windows of 25, 50 and 100 kbp, and nucleosome occupancy examined. By 10 min, alternating enriched and depleted domains were seen at all window sizes; by 30 min most of these further evolved (Additional file 8A) and depleted profiles predominated (also reproducible between replicates; Additional file 8B). In other words, a gradual spreading of nucleosome-depleted domains was observed, and this appeared to be nucleated by the hotspots seen at 10 min (many also engulfing DNase-hypersensitive sites, especially by 30 min post-stimulation; Additional file 8C).

**Figure 4:**
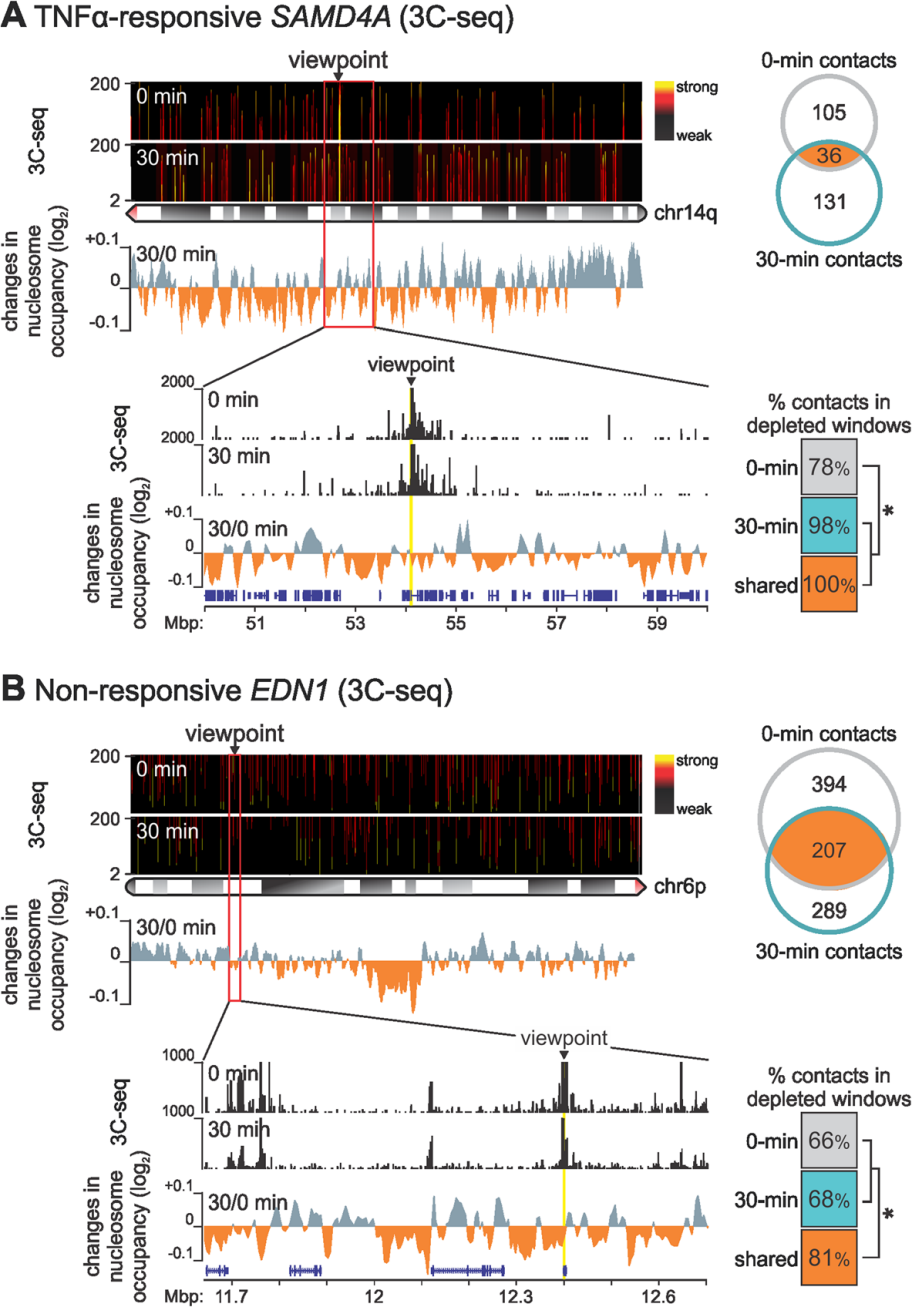
**Changes in nucleosome positioning affect higher-order structure. (** **A)** High-confidence contacts (*P* <0.05; determined using 3C-seq 0 or 30 min post-stimulation) made by the transcription start site (TSS; *arrowhead*) of TNFα-responsive *SAMD4A* with parts of the long arm of chromosome 14 (*ideogram*) are depicted as a domainogram (*y-axis* - contacts visualized in 2- to 200-kbp sliding windows). Most contacts are unique for each time point (Venn diagram). The magnified region (*red rectangle*) compares 3C-seq contacts (*y-axis* - reads per million) to changes in nucleosome occupancy (determined as in Figure 2). The table (*bottom right*) gives the fraction of 3C contacts embedded in nucleosome-depleted windows at 0 or 30 min, or shared at both times; a significant increase is seen for 30-min and shared contacts (*P <0.05; Fisher’s exact test). **(B)** Details as in panel (A), for the non-responsive *EDN1* TSS (*arrowhead*) on the long arm of chromosome 6 (*ideogram*). Almost 40% of high-confidence contacts persist from 0 to 30 min (Venn diagram), and are significantly associated with nucleosome-depleted 5-kbp windows (*P <0.05; Fisher’s exact test). 3C-seq, chromosome conformation capture coupled to deep sequencing; TNFα, tumour necrosis factor alpha.

To relate changes in nucleosome occupancy to those in DNA conformation, we performed 3C-seq at 0 and 30 min post-stimulation [21] using the TSSs of TNFα-responsive *SAMD4A* and constitutively expressed *ΕDN1* as viewpoints. For the *SAMD4A* TSS, we showed previously that stimulation induces development of new contacts throughout the genome [18]; here we focus only on the more abundant intra-chromosomal contacts. At 0 min, *SAMD4A* contacts were scattered throughout the chromosome arm, and after 30 min new ones developed (Figure 4A, *top*). Of the 167 most frequently seen 30-min contacts, 131 formed *de novo* upon TNFα treatment. When correlated with changes in nucleosome occupancy (in 5-kbp windows, as in Figure 2), we found essentially all 30-min and ‘shared’ contacts embedded in nucleosome-depleted windows (significantly more than 0-min contacts; Figure 4A).

By contrast, the *EDN1* TSS formed fewer new contacts upon stimulation (of the 496 most frequent 30-min contacts 42% were also see at 0 min; Figure 4B, *top*). Moreover, significantly more shared contacts correlated with nucleosome-depleted windows (compared to 0- or 30-min specific ones; Figure 4B). Closer inspection of the two loci shows that contacts (in accord with obtained chromatin interaction analysis by paired-end tag sequencing data [18]) do not form randomly between ‘nucleosome-free’ regions, but rather share particular features (that is, NF-κB binding, H3K4me1 enrichment and transcriptional activity; Additional file 9).

## Discussion

We addressed the question: how does TNFα stimulation reshape the chromatin landscape as it establishes the immediate-early proinflammatory transcriptional programme? The cytokine signals through NF-κB [13], and one might envisage that the factor first binds in the vicinity of regulatory elements to induce repositioning of nucleosomes locally. This would then facilitate transcriptional initiation by RNA polymerase, and would in turn open up the bodies of TNFα-responsive genes as polymerases elongate through them [32,33]. However, changes observed here cannot be reconciled with this scenario.

First, we saw hotspots of nucleosome depletion 10 min post-stimulation (Additional file 8Α), before detectable NF-κB binding to cognate sites (Additional file 6Α). Although there were approximately 1,300 NF-κB binding peaks in nucleosome-depleted windows after 30 min, most bound NF-κB was not embedded in kilobase pair-long depleted regions (Figure 3A). This also fits with the distribution of typical NF-κB motifs (5′-GGRRNNYYCC-3′): out of >550,000 sites found genome-wide, only 60,000 and 250,000 were embedded in windows depleted of nucleosomes after 10 and 30 min, respectively (with 28,000 being shared and very few being occupied; Figure 3A). It follows that NF-κB binding is highly selective; the first transcription factor complexes to enter nuclei (between 10 and 15 min) must preferentially bind to a small subset of primed domains depleted of nucleosomes, harbouring the highest affinity sites - probably within the critical enhancers that regulate the ensuing cascade and/or on particular *Alu* repeats [24]. This is reminiscent of a subset of NF-κB dimers in macrophages selectively binding to already-accessible chromatin segments where partner regulators constitutively bind [34] - which raises the question of what the endothelial-specific NF-κB partners might be.

Second, results cannot be reconciled with the idea that transcription through nucleosomes by pioneering elongating RNAPs is solely responsible for changes in chromatin structure. Nucleosomes in long TNFα-responsive genes are repositioned throughout, well before elongating polymerases have transversed their full length (Figure 2). Then, what molecular mechanism might drive repositioning at sites many kilobase pairs away from a bound NF-κB or a pioneering polymerase? We can suggest some possibilities that might act singly, or in concert. For example, an effector other than NF-κB might be responsible for priming; then, NF-κB (and/or another effector) could induce chromatin remodelling enzymes to act throughout the surrounding locale - perhaps a chromatin loop or cluster of loops in a topological domain attached to a transcriptional hot spot [35]. Alternatively, transcription could generate supercoiling that remodels one such loop (or cluster of loops) within a topological domain [36]. Lastly, polymerases other than pioneers on responsive genes could drive repositioning - perhaps ones generating enhancer RNAs (like in Additional file 6B) [37]. This is supported by the presence of NF-κB clusters bound within gene bodies at sites marked by histone marks and transcripts characteristic of enhancers; these overlap ‘super-enhancers’ previously mapped in HUVECs [38] that also show decreased nucleosome density post-stimulation (see examples in Figure 3C and Additional file 6B).

Third, nucleosome repositioning has traditionally been viewed as a local phenomenon, but we detect occupancy changes throughout megabase pair-long segments (see chromosomes 4 and 14 in Additional file 8). (Note that, using semi-quantitative Western blotting with antibodies targeting histones H3 and H4, we verified TNFα stimulation does not affect global histone levels; data not shown.) Using 3C-seq, we confirmed the intuition that changes in nucleosome positioning around two megabase pair-long chromosomal loci go hand-in-hand with the development of contacts in three-dimensional nuclear space. Interestingly, a subset of recorded 3C contacts - which predominantly form between regulatory *cis*-modules [39,40] marked by NF-κB and characteristic histone modifications (Additional file 9) - persist throughout the transition from the unstimulated to the TNFα-stimulated state (Figure 4). This is consistent with pre-looped chromatin facilitating responses to extra-cellular cues [41], and can now be explained also at the level of nucleosomal organization.

## Conclusions

Collectively, our data point to TNFα triggering chromatin priming so that most nucleosomes are repositioned independently of NF-κB binding and/or polymerases elongating through responsive genes. This effect is a prelude to the ensuing proinflammatory programme, and it occurs both locally (at the gene level) as well as at considerable distances from, what have hitherto been considered, the major nucleating sites to affect large chromosomal segments. Finally, although ‘topological domains’ may constitute invariant building blocks within chromatin [41–43], an underlying and plastic network of interactions within a domain must affect DNA accessibility to polymerases, ultimately allowing the rapid transitions that occur as different sets of genes become active and inactive and the inflammatory cascade unfolds [15,16]. Of course, the molecular machines responsible for priming, their interplay with NF-κB, and the potential role of other factors (like histone H1 eviction or activity of topoisomerases) need be addressed in light of these findings.

## Methods

### Cell culture

HUVECs from pooled donors (Lonza, Cologne, Germany) were grown to 80% to 90% confluence in endothelial basal medium 2-MV with supplements (EBM; Lonza) and 5% foetal bovine serum (FBS); starved for 16 to 18 h in EBM +0.5% FBS; treated with TNFα (10 ng/ml; Peprotech, Hamburg, Germany); and harvested 0, 10 or 30 min post-stimulation.

### Isolation of mononucleosomes, sequencing and mapping

Approximately 5 × 10^6^ HUVECs stimulated with TNFα for 0, 10 or 30 min were digested (3 min at 37°C) with 750 units of micrococcal nuclease (MNase; Sigma-Aldrich, Seelze, Germany). Mononucleosomal DNA was isolated following separation on 1.3% agarose gels using glass beads (Qiagen, Hilden, Germany), and average fragment lengths determined using a 2100 Bioanalyzer (Agilent). Libraries were generated using the NEBNext DNA Library Prep Master Mix Kit (New England Biolabs, Ipswich, USA) and paired-end (2 × 50-bp) sequenced on a HiSeq2000 platform (Illumina, Essex, UK) to comparable depths (that is, 181, 185 and 187 million reads for 0, 10 and 30 min samples, respectively). Obtained reads were processed using the toolkits FastQC [44] and FASTX [45], mapped to hg19 using Bowtie [46].

### MNase-seq analysis

Different peak-calling algorithms were applied depending on the downstream application. For Additional file 4 the Peak Predictor/GeneTrack package [30] was used. For motif analyses, as well as Gene and Genome Ontology profiling (Additional file 1 and Table 1), the HOMER software package [47] and findPeaks 3.1 [23] were applied (adjusting fragment size to that determined using the Bioanalyzer with the following settings: −*style factor –size 147 –minDist 1 –F 0 –L 0 –C 0*). When comparing two or more datasets, the *getDifferentialPeaks* or *mergePeaks* scripts were used. For visualization, tag directories of mapped reads were generated and .bedGraph files produced using the *makeUCSCfile* (for raw reads) or *pos2bed.pl* (for peaks and other BED-formatted files) scripts; tracks were then visualized with the UCSC Genome browser [48]. Both known and *de novo* motif analyses were performed with *findMotifsGenome.pl* using standard settings and the repeat-masked hg19 genome build. All peak annotations, including histograms, were generated with *annotatePeaks.pl*, and graphs plotted in R [49] with a smoothing spline of 0.2.

Differences in nucleosome positioning between any two time-points (0- compared to 10- or 30-min datasets) were elucidated statistically using a novel Neyman-Pearson ‘normalized log-likelihood-ratio’ analysis. Chromosomes 1-X were divided in *n* non-overlapping windows *w*_1,_*w*_2,_ … *w*_*n*_ of a constant size |*w*_i_|. In a pre-processing step, MNase-seq data files containing read positions at *t*_1_ and *t*_2_ were used to compile datasets *R* = (*r*_1_, *r*_2_, … *r*_*n*_) and *S* = (*s*_1_, *s*_2_, … *s*_*n*_); *r*_*i*_ and *s*_*i*_ are the read counts in each *w*_*i*_ observed under treatments *t*_1_ and *t*_2_, respectively. Then hypotheses *H*_1_ and *H*_2_ were tested by computing a log-likelihood-ratio *Q* according to:

$$ Q= \log \frac{R}{S}=\left({q}_1,{q}_2\;....\kern0.5em {q}_n\right);\kern1em {q}_i=\frac{r_i}{s_i}. $$

This set of log-likelihood-ratio values has a mean of ${Q}_{mean}=\frac{1}{n}{\displaystyle \sum_{i=1}^n{q}_i}$ and a normalized distribution ||*Q*|| = *Q* - *Q*_*mean*_. It follows that ||*q*_*i*_|| values are centred on zero. The null hypothesis is then that all observed *q*_*i*_-values from regions that were transcriptionally inert (assessed using RNA-seq data) were due to random fluctuations and not caused by treatments *t*_1_ and *t*_2_. The normalized cumulative distribution *N*_*cum*_ was used to determine a p-value p(||*q*_*i*_||) for ||*q*_*i*_|| ≥0 according to:

$$ p\left(\left|\left|{q}_i\right|\right|\right)=1-{N}_{cum}\left(\left|\left|{q}_i\right|\right|\right) $$

Thus, the smaller *p*(||*q*_*i*_||) is, the lower the probability that the ratio ||*q*_*i*_|| is merely due to a stochastic fluctuation of read counts.

### Chromosome conformation capture

Nuclei were harvested after 0 or 30 min of TNFα stimulation, cross-linked in 1% paraformaldehyde (PFA; Electron Microscopy Science, Munich, Germany), and processed as described [21] using *Apo*I as the primary restriction endonuclease. Following sequencing on a HiSeq2000 platform (Illumina; approximately 2 × 10^7^ reads), data were analysed using the r3Cseq pipeline [50]. The domainogram in Figure 4 was generated using the top 167 *cis*-contacts on chromosome 14 (on which the viewpoint lies) using publicly available software [51]. In brief, 3C-seq reads are made binary and relative enrichments calculated using sliding windows compared to a randomized background made up of 3,000 fragment ends. Data permutation is then used to determine a threshold of <0.01 false discovery rate (FDR); windows exceeding this threshold are scored as interacting.

### Chromatin immunoprecipitation and ChIP-seq analysis

Approximately 10^7^ HUVECs were cross-linked (using 1% PFA for 10 min, preceded by 25 min in 10 mM ethyl-glycol-*bis*-succinimidylsuccinate at room temperature, as described previously [18]) 0, 10 or 30 min after TNFα stimulation; chromatin was fragmented by sonication (Bioruptor; Diagenode, Liège, Belgium); then immunoprecipitation was carried out using a rat monoclonal against phospho-Ser2 in the C-terminal domain of the largest subunit of RNA polymerase II (3E10 [52]; a gift from Dirk Eick, Helmholtz Institute, Munich, Germany) or a rabbit polyclonal against the full-length p65 subunit of NF-κB (39369, Active motif) on aliquots of approximately 25 μg chromatin. Immunoprecipitated complexes were washed and eluted using the ChIP-It-Express kit (Active motif, Rixensart, Belgium).

For qPCR analysis, a Rotor-Gene 3000 cycler (Qiagen) and Platinum SYBR Green qPCR SuperMix-UDG (Invitrogen, Darmstadt, Germany) were used. Following incubation at 50°C for 2 min to activate the qPCR mix, and 95°C for 5 min to denature templates, reactions were carried out for 40 cycles at 95°C for 15 s, and 60°C for 50 s. PCR primers were designed via Primer3Plus [53] using *qPCR* settings with an optimal length of 20 to 22 nucleotides, a Tm of 62°C, targeting 100 to 200 bp. The presence of single amplimers was confirmed by melting-curve analysis, and data were analysed to obtain enrichments relative to input. *P* values (two-tailed) from unpaired Student’s *t*-tests [54] were considered significant when <0.05.

For deep sequencing, previous (0- and 30-min [18]) and newly generated (10-min) p65 ChIP-seq data were aligned to hg18 and signal peaks detected using MACS [55]. This allowed 68, 214 and 8,583 high-confidence p65-binding events to be detected for 0, 10 and 30 min respectively (FDR ≤0.01, peak height ≥20 reads/million). Peaks were correlated to publicly available ENCODE Hidden Markov chromatin models and HUVEC ChIP-seq data (H3K27ac: GSM733691; H3K4me1: GSM733690 [31,56]) and annotated against RefSeq genomic features (TSS, exon, intron, intergenic region).

### Total RNA sequencing and analysis

Total RNA was isolated from 0.5 × 10^6^ HUVECs stimulated with TNFα for 0, 10 or 30 min using TRIzol (Invitrogen), treated with RQ1 DNase (1 unit/μg RNA, 37°C, 45 min; Promega, Leiden, Netherlands), depleted of rRNA (RiboMinus; Epicentre, Madison, USA), chemically fragmented to approximately 350 nucleotides, and cDNA generated using random hexamers as primers (according to the True-seq protocol; Illumina). Adapters were then ligated to cDNA molecules, and libraries sequenced (Illumina HiSeq2000 platform; 100-bp paired-end reads; around 120 × 10^6^ read-pairs per sample). Raw reads were then mapped to hg18 using TopHat [57] and reads aligning to RefSeq gene models were counted using the HTseq package [58]. Statistical analysis of differentially expressed genes was performed with the DESeq Bioconductor package [59] (asking for >100 reads per gene, and for a >0.6, <−0.6, or ±0.01 log_2_ fold-change for up-regulated, down-regulated or constitutively expressed genes, respectively; Additional file 3).

### Immunofluorescence

HUVECs grown on coverslips etched with hydrofluoric acid were fixed with 4% PFA (Electron Microscopy Science) in phosphate-buffered saline (PBS; 20 min, 20°C), washed once in PBS (5 min, 20°C), permeabilized using 0.5% Triton X-100 in PBS (5 min, 20°C) and blocked with 1% bovine serum albumin (BSA) in PBS (Sigma-Aldrich; 45 min, 20°C). Phosphorylated (at Ser536) p65 was detected using a rabbit monoclonal antibody (1:500 dilution, 0.5% BSA in PBS; #04-1000, Millipore, Nottingham, UK) and Alexa488-conjugated donkey anti-rabbit AffinityPure F(ab’)2 Fragment (1.5 μg/ml; Jackson ImmunoResearch, Maine, USA). After DAPI counter-staining, images were collected on a Leica DMI6000 B widefield microscope and analysed using ImageJ [60]; nuclei were encircled, the mean intensity calculated per area, and nuclear fluorescence (arbitrary units) calculated by subtracting the background (measured as the minimum intensity in the image).

### Data availability

MNase-seq raw data are available at the GEO database under accession number [GEO: GSE53343], while 3C-seq, p65 ChIP-seq and total (ribo-depleted) RNA-seq data generated here can be accessed at the SRA archive under accession number [SRA: SRP044729].

## Additional files

Additional file 1:
**Reproducibility, motif/GO term analysis and NF-κB translocation.**
Additional file 2:
**TNFα-regulated genes and expression levels.**
Additional file 3:
**Differential gene expression (30- compared to 0-min levels).** Total (ribo-depleted) RNA‐seq data were obtained 0 and 30 min post‐stimulation, mapped to the reference genome, and analysed. All RefSeq human genes are listed with associated mean (base mean) number of mapped reads (normalized to library size), and log2 fold-changes resulting from the 30- versus 0-min comparison. Additional file 4:
**TNFα induces repositioning throughout long responsive genes.**
Additional file 5:
**Reproducibility of repositioning profiles along long genes.**
Additional file 6:
**Characteristics of NF-κB binding events.**
Additional file 7:
**Comparison of inter- and intra-genic NF-κB binding events.**
Additional file 8:
**Multi-scale domains and changes in nucleosomal organization.**
Additional file 9:
**Chromatin interaction analysis by paired-end tag sequencing supports 3C-seq data along two chromosomal loci.**

## Abbreviations

3C-seq: chromosome conformation capture coupled to deep sequencing
bp: base pair
BSA: bovine serum albumin
ChIP-seq: chromatin immunoprecipitation coupled to high-throughput sequencing
EBM: endothelial basal medium
FBS: foetal bovine serum
FDR: false discovery rate
kbp: kilobase pair
MNase-seq: micrococcal nuclease digestion followed by sequencing
NF-κB: nuclear factor kappa-B
PFA: paraformaldehyde
PBS: phosphate-buffered saline
RNAP: RNA polymerase
RNA-seq: sequencing of total RNA
TNFα: tumour necrosis factor alpha
TSS: transcription start site
TTS: transcription termination site
