# Supplementary material for: TNFα signalling primes chromatin for NF-κB binding and induces rapid and widespread nucleosome repositioning
Source: Genome Biol. 2014 Dec 3;15(12):536. doi: 10.1186/s13059-014-0536-6 (PMC4268828; doi:10.1186/s13059-014-0536-6)
Supplement: Additional file 1: — Reproducibility, motif/GO term analysis and NF-κB translocation. [file 13059_2014_536_MOESM1_ESM.pdf]

Additional File 1

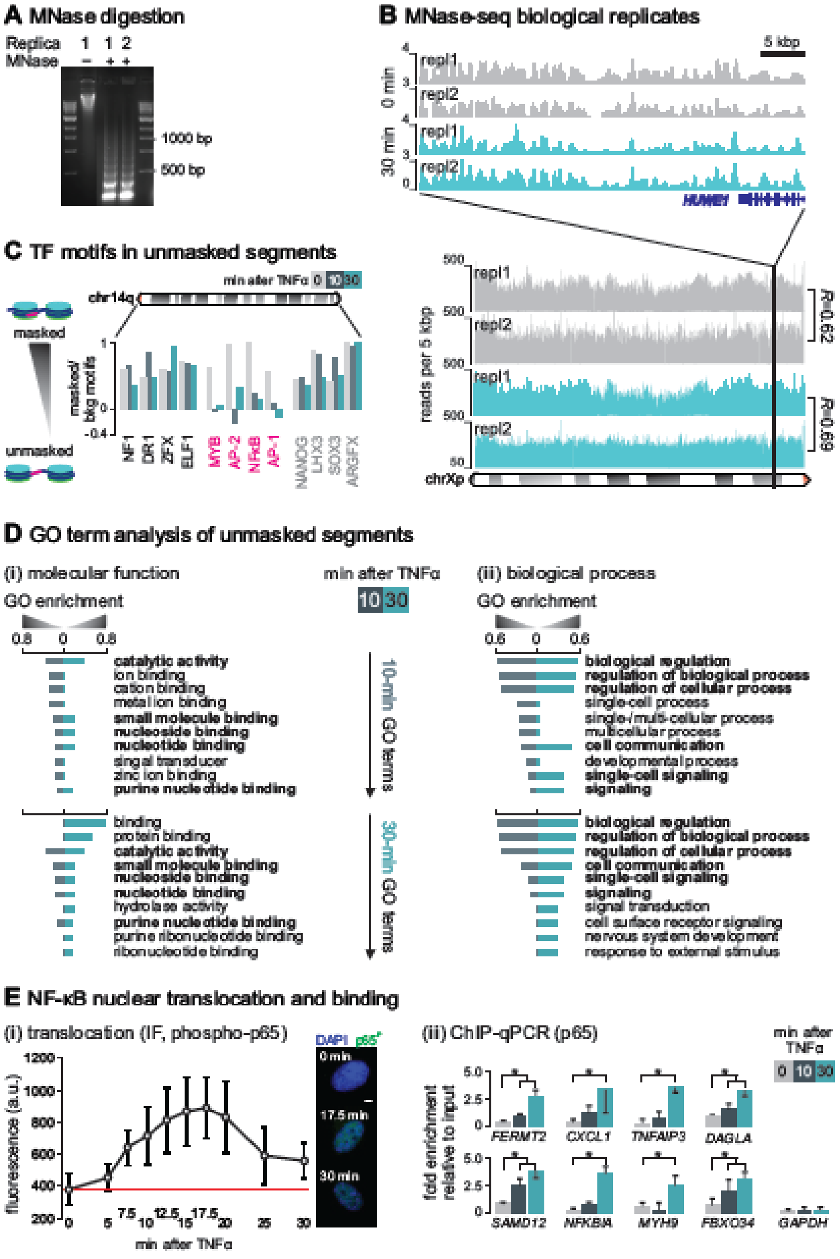

Additional File 1 | Reproducibility, motif/GO term analysis, and NF-κB translocation. (**A**) Typical gel-electrophoresis profiles obtained with two biological replicates (*1*, *2*) and treatments ± MNase 30 min post-stimulation (molecular-weight markers, with the 1000- and 500-bp bands indicated, flank test samples). (**B**) Reproducibility of MNase-seq. Browser views of constitutively-expressed *HUWE1* (*top*) show similar MNase-seq profiles between replicates at 0 and 30 min (expressed in "reads per million"). This is exemplified using the long arm of chromosome X (*ideogram*), where the sum of raw MNase-seq reads in 5-kbp non-overlapping windows is shown. Spearman's correlation values for each replicate pair are also presented. (**C**) Unmasked regions on the long arm of chromosome 14 (*ideogram*) encode motifs of transcription factors (TFs) involved in TNFα signaling. The top 4 motifs known to bind constitutively-expressed (*black*), TNFα-induced (*red*), or non-expressed TFs in HUVECs (*grey*) are presented. The observed frequency of a motif in nucleosome-covered regions relative to a control set of randomly-selected sequences (background: "bkg") is expressed as a ratio (i.e. [nucleosome-masked motifs – random motifs]/nucleosome-masked motifs). Reduced ratios reflect motif unmasking (*cartoon*). (**D**) Gene Ontology (GO) terms associated with unmasked segments. Regions masked by a nucleosome only at 0 min were identified. Then, the ten top GO terms associated with (i) "molecular function" or (ii) "biological process" terms in regions unmasked at 10 (*top*) or 30 min (*bottom*) were selected, and enrichments in the 10- (*grey*) and 30-min (*green*) datasets plotted (scale gives enrichment over background**, and** GO terms shared between 10- and 30-min sets are shown in bold). (**E**) Features of NF-κB translocation to the nucleus and binding. (**i**) Activated (phosphorylated at Serine 536 [18]) p65 was localized by immune-fluorescence 0-30 min post-stimulation, and total nuclear fluorescence levels (a.u.) recorded and presented as box plots; *red line*: 0-min levels. Typical images are shown; *bar*: 2 μm. (**ii**) ChIP-qPCR was performed 0, 10, and 30 min post-stimulation using an antibody targeting p65 and amplicons carrying NF-κB binding sites upstream the TSS of 8 responsive genes (*GAPDH* serves as a control). *: significantly different from 0 min; $P<0.05$, two-tailed, unpaired Student's *t*-test.
