# Supplementary material for: TNFα signalling primes chromatin for NF-κB binding and induces rapid and widespread nucleosome repositioning
Source: Genome Biol. 2014 Dec 3;15(12):536. doi: 10.1186/s13059-014-0536-6 (PMC4268828; doi:10.1186/s13059-014-0536-6)
Supplement: Additional file 2: — TNFα-regulated genes and expression levels. [file 13059_2014_536_MOESM2_ESM.pdf]

Additional File 2

A RNA-seq (30- v 0-min post-TNFα)

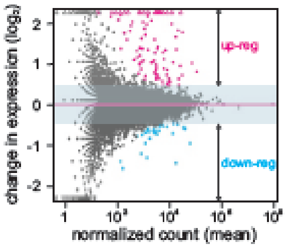

gene	0-min	30-min	fold-ratio	gene	0-min	30-min	fold-ratio
CXCL3	4.79	1591.20	8.10	*CCDC88B*	116.92	62.60	-0.89
CCL20	0.00	146.08	7.20	*MRPL54*	1175.96	587.44	-1.00
CXCL2	38.34	5565.56	7.14	*RPL27A*	48693.17	24301.05	-1.00
SELE	126.51	10790.96	6.40	*ETV4*	115.01	56.34	-1.02
IL8	27.79	2257.94	6.29	*HOXA6*	139.93	65.73	-1.08
CXCL1	92.01	5085.59	5.77	*NDUFB6*	898.02	371.45	-1.27
CD69	9.58	390.24	5.21	*ARL2*	1458.68	563.44	-1.37
ZFP36	486.86	15924.54	5.03	*MYEOV*	161.97	58.43	-1.46
TNFAIP3	292.31	5566.60	4.25	*RPLP2*	16982.76	5948.49	-1.51
NFKBIA	994.81	15021.99	3.92	*MX1*	204.14	64.69	-1.64

B p65-proximal genes

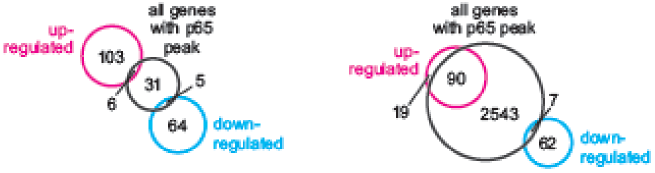

C Browser views of typical genes

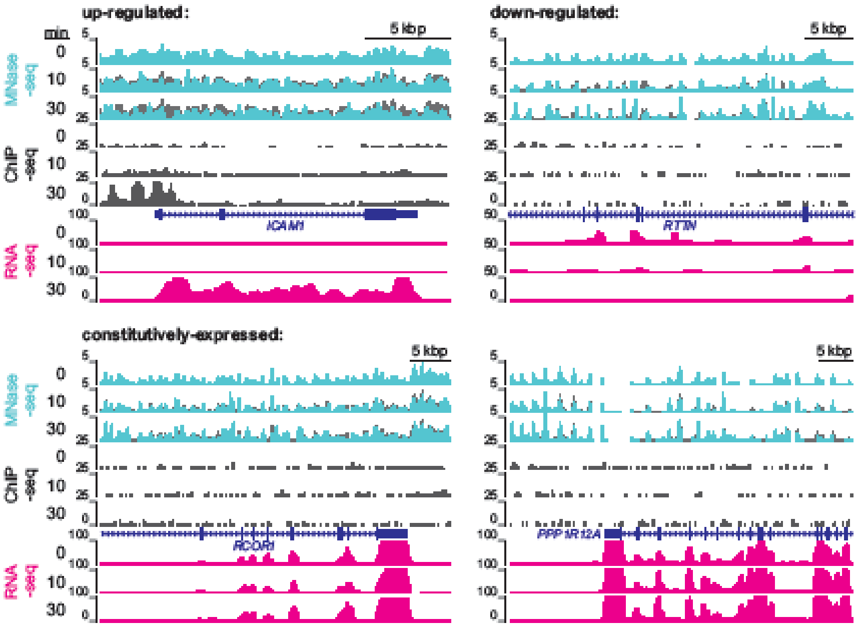

Additional File 2 | TNFα-regulated genes and expression levels. (A) Changes in gene expression assessed by RNA-seq. Total RNA was isolated from HUVECs 0 or 30 min post-stimulation, rRNA-depleted, deep-sequenced, and numbers of reads mapping to genes (normalized to library size) determined. *Left*: Read numbers for each gene (single dot) are plotted against change in expression; up-/down-regulated genes (*red* and *blue* dots, respectively) were selected using a ±0.6 $\log_2$ (~1.5-fold) and ≥100 reads/gene cutoff. *Right*: details for the 10 most up-/down-regulated genes after 30 min (raw read counts per gene model at the relevant times, and fold change between the two). (B) Differentially-regulated genes proximal to p65 peaks. Venn diagrams show the number of RefSeq genes that were up-/down-regulated 30 min post-stimulation (*red*/*blue*; from panel A) and marked by p65 peaks lying within 2 kbp of the gene after 10 or 30 min. (C) Browser views (*y-axis*: reads per million) illustrating data for typical up-/down-regulated and constitutively-expressed genes obtained by MNase-seq (0-min levels in *grey* underlie 10- and 30-min in *green* to facilitate comparison), p65 ChIP-seq (*dark grey*), and RNA-seq (*magenta*) at the same time-points.
