# Supplementary material for: TNFα signalling primes chromatin for NF-κB binding and induces rapid and widespread nucleosome repositioning
Source: Genome Biol. 2014 Dec 3;15(12):536. doi: 10.1186/s13059-014-0536-6 (PMC4268828; doi:10.1186/s13059-014-0536-6)
Supplement: Additional file 4: — TNFα induces repositioning throughout long responsive genes. [file 13059_2014_536_MOESM4_ESM.pdf]

Additional File 4

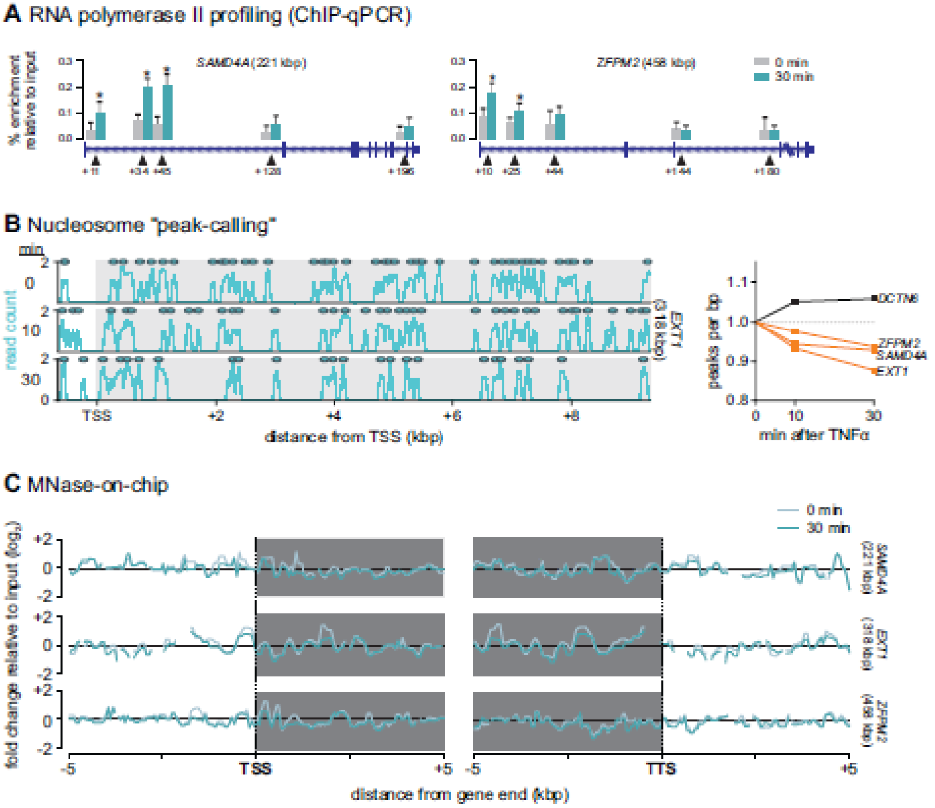

Additional file 4 | TNFα induces repositioning throughout long responsive genes. (A) ChIP-qPCR (using an antibody targeting hyper-phosphorylated Ser2 in heptad repeats of the C-terminal domain of *RPB1* [50], and primers targeting the sites indicated by arrowheads) confirms that after 30 min pioneering RNA polymerases are only detected close to 5' ends of 221-kbp *SAMD4A* and 458-kbp *ZFPM2*. *: significantly different from 0-min levels (P<0.05, unpaired two-tailed Student's t-test). (B) Identification of single-nucleosome positions illustrates a drop in occupancy. *Left*: A 12-kbp view around the TSS of TNFα-responsive *EXT1* showing MNase-seq profiles at different times. Ovals (*green*) mark nucleosome positions called using "Peak Predictor" [30] (threshold of 1.0); 41, 38, and 24 nucleosomes are called in this region at 0, 10, and 30 min, respectively. *Right*: Peaks obtained with four genes after 10 and 30 min were normalized relative to gene length and 0-min peak number; peak depletion seen at the TSS extends throughout responsive *EXT1*, *SAMD4A*, and *ZFPM2*, but not non-responsive *DCTN6*. (C) Changes in nucleosome occupancy on responsive *SAMD4A*, *EXT1*, and *ZFPM2* assessed using "MNase-on-chip". Mononucleosomal DNA (prepared as for MNase-seq) and randomly-sheared genomic DNA were labelled, mixed, and applied to a microarray bearing overlapping probes spanning the genes. After normalization, ratios reflect increased/reduced occupancy (combined results from 3 biological replicates were smoothed using a 200-bp sliding window) at 0 (*grey*) and 30 min (*green*). For clarity, only ±5 kbp around TSSs and TTSs are shown.
