# Supplementary material for: TNFα signalling primes chromatin for NF-κB binding and induces rapid and widespread nucleosome repositioning
Source: Genome Biol. 2014 Dec 3;15(12):536. doi: 10.1186/s13059-014-0536-6 (PMC4268828; doi:10.1186/s13059-014-0536-6)
Supplement: Additional file 5: — Reproducibility of repositioning profiles along long genes. [file 13059_2014_536_MOESM5_ESM.pdf]

Additional File 5

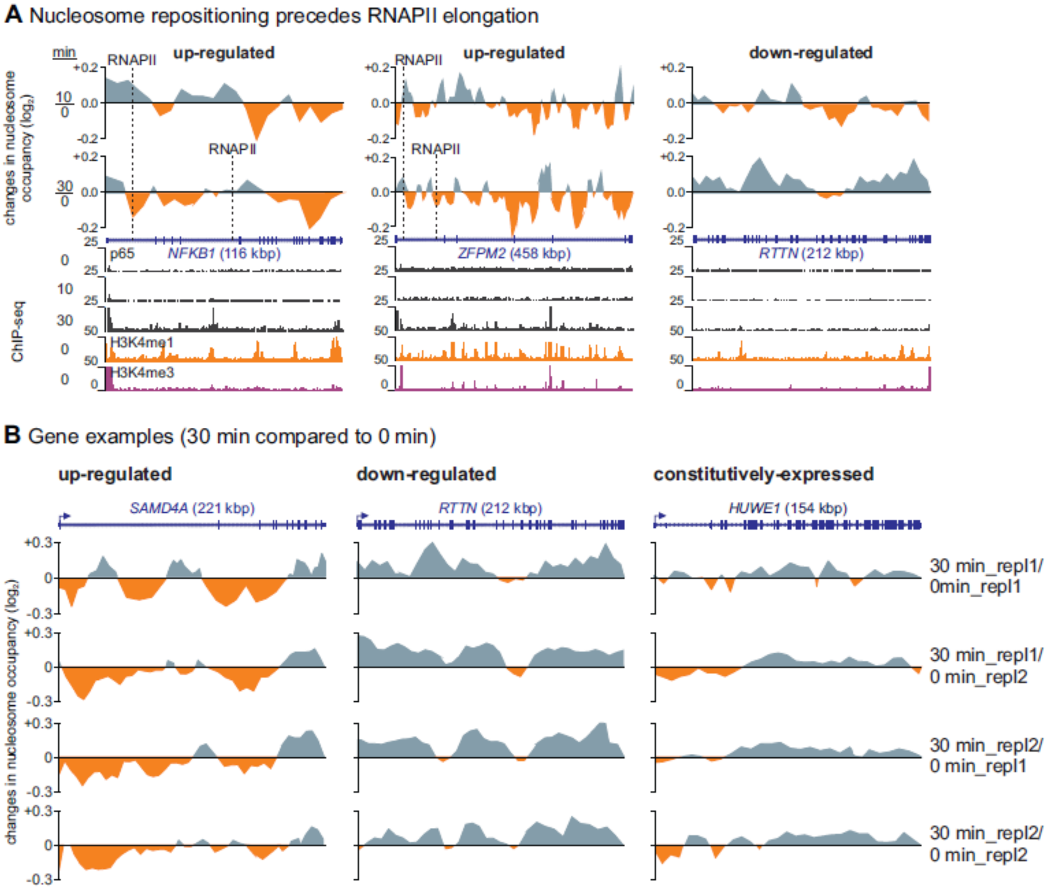

Additional File 5 | Changes in nucleosome occupancy along genes. (**A**) Brower views showing (log_2 fold) changes in nucleosome occupancy at 10 or 30 min (determined as in **Figure 2**; enrichment: *grey*, depletion: *orange*) along three long genes. Tracks for p65 (*black*), H3K4me1 (*orange*), and H3K4me3 (*purple*) ChIP-seq are shown below (*y-axis*: reads/million). (**B**) Reproducibility of observed changes in nucleosome occupancy (determined as in **Figure 2**). 0- and 30-min MNase-seq data from two biological replicates ("repl1" and "repl2"), compared in all pairwise combinations, yield similar enrichment/depletion profiles; typical examples of up-, down-regulated, and constitutively-expressed genes are shown.
