# Supplementary material for: TNFα signalling primes chromatin for NF-κB binding and induces rapid and widespread nucleosome repositioning
Source: Genome Biol. 2014 Dec 3;15(12):536. doi: 10.1186/s13059-014-0536-6 (PMC4268828; doi:10.1186/s13059-014-0536-6)
Supplement: Additional file 6: — Characteristics of NF-κB binding events. [file 13059_2014_536_MOESM6_ESM.pdf]

Additional File 6

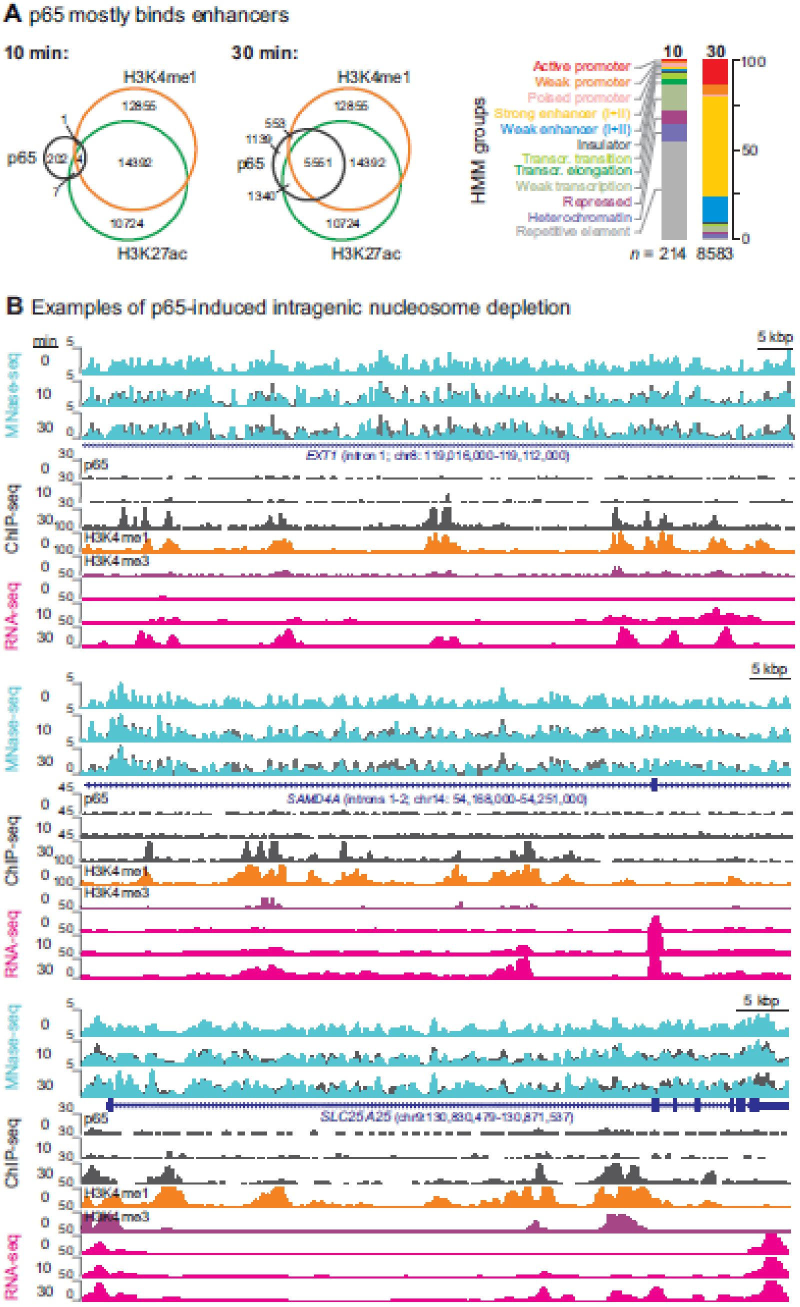

Additional File 6 | Characteristics of p65 binding genome-wide. (**A**) Sites binding p65 carry histone marks characteristic of enhancers. Strong peaks of p65 binding 10 or 30 min post-stimulation were selected (≥20 reads/million; FDR <0.01; *n*=214 and 8,583, respectively). *Left/middle*: Venn diagrams showing that ~65% of 30-min peaks carry histone modifications typical of enhancers (i.e., H3K4me1, H3K27ac; determined using HUVEC ENCODE data [31]). *Right*: HUVEC chromatin, segmented using epigenetic marks and hidden Markov models (HMM [51]), was compared to 10- and 30-min p65 binding profiles; most 10-min peaks overlap repetitive elements, while >75% 30-min p65 sites are embedded in regions characterized as "strong" enhancers. (**B**) Examples of intra-genic p65 peaks. Browser tracks illustrate MNase-seq (0-min levels in *grey* underlie 10- and 30-min in *green* to facilitate comparison), p65 (*black*) or H3K4me1/3 ChIP-seq (*orange* and *purple*, respectively), and RNA-seq data (*magenta*) in the introns of TNFα-responsive genes *EXT1*, *SAMD4A*, and *SLC25A25*.
