# Supplementary material for: TNFα signalling primes chromatin for NF-κB binding and induces rapid and widespread nucleosome repositioning
Source: Genome Biol. 2014 Dec 3;15(12):536. doi: 10.1186/s13059-014-0536-6 (PMC4268828; doi:10.1186/s13059-014-0536-6)
Supplement: Additional file 7: — Comparison of inter- and intra-genic NF-κB binding events. [file 13059_2014_536_MOESM7_ESM.pdf]

Additional File 7

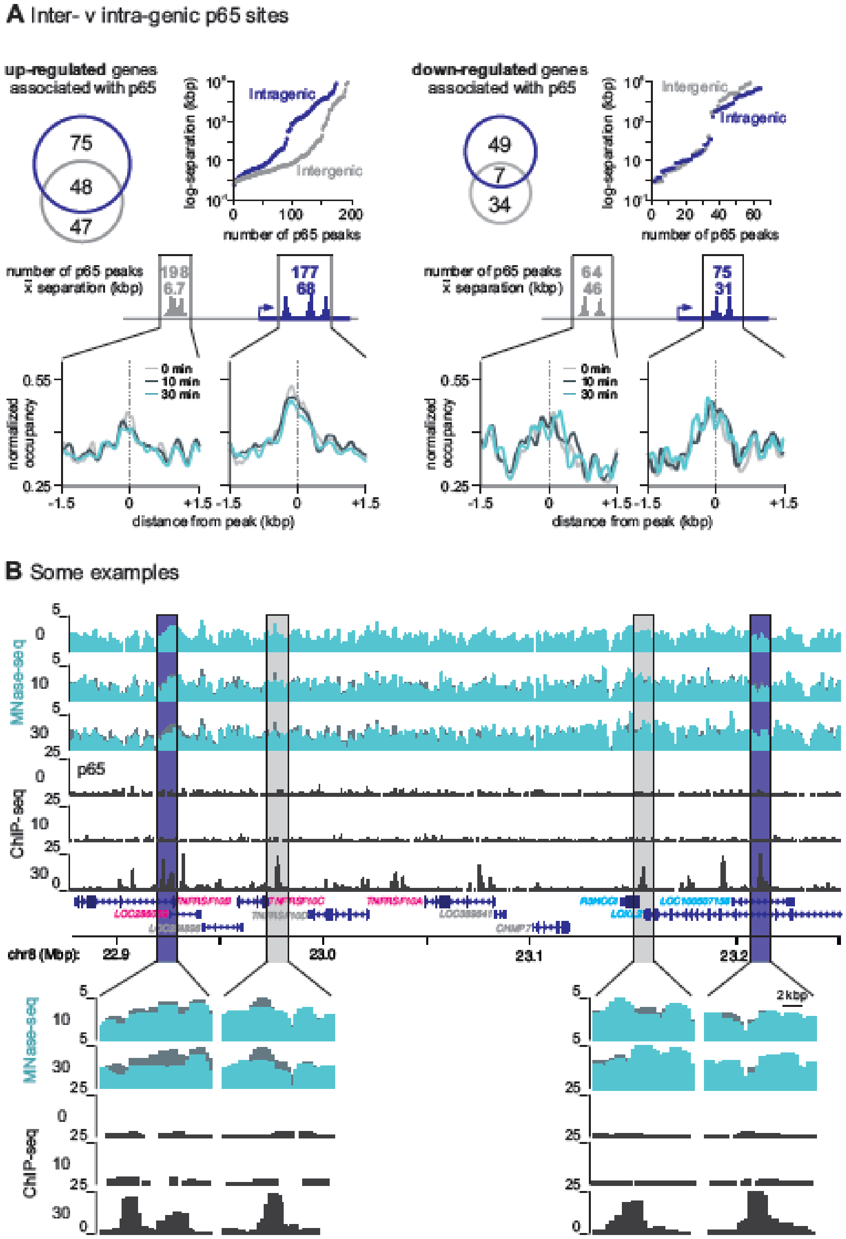

Additional File 7 | Comparison of inter- and intra-genic NF-κB binding events. (**A**) Genes were classified as up- (*left*) or down-regulated (*right*) without a read cutoff (to include more genes in the analysis), and p65 peaks in/around them as intra- (*blue*) or inter-genic (*grey*), respectively. *Top*: Venn diagrams show the number of genes associated with ≥1 p65 inter-/intra-genic peak; cumulative plots show minimum separations between peaks. *Middle*: Cartoons illustrate the numbers and median separations of inter- (*grey*) and intra-genic (*blue*) p65 peaks in each gene subset. *Bottom*: Normalized nucleosome occupancies around p65 peaks (calculated by summing occupancies at 50-bp intervals) at 0, 10, and 30 min. (**B**) Examples of intra- (*highlighted blue*) and inter-genic (*highlighted grey*) p65 peaks along a typical locus on chromosome 8. Browser tracks illustrate "reads per million" obtained by MNase-seq (0-min levels in *grey* underlie 10- and 30-min in *green* to facilitate comparison) and p65 ChIP-seq (*black*). Magnifications of nucleosome occupancy around typical p65 binding peaks are shown below.
