# Supplementary material for: TNFα signalling primes chromatin for NF-κB binding and induces rapid and widespread nucleosome repositioning
Source: Genome Biol. 2014 Dec 3;15(12):536. doi: 10.1186/s13059-014-0536-6 (PMC4268828; doi:10.1186/s13059-014-0536-6)
Supplement: Additional file 8: — Multi-scale domains and changes in nucleosomal organization. [file 13059_2014_536_MOESM8_ESM.pdf]

Additional File 8

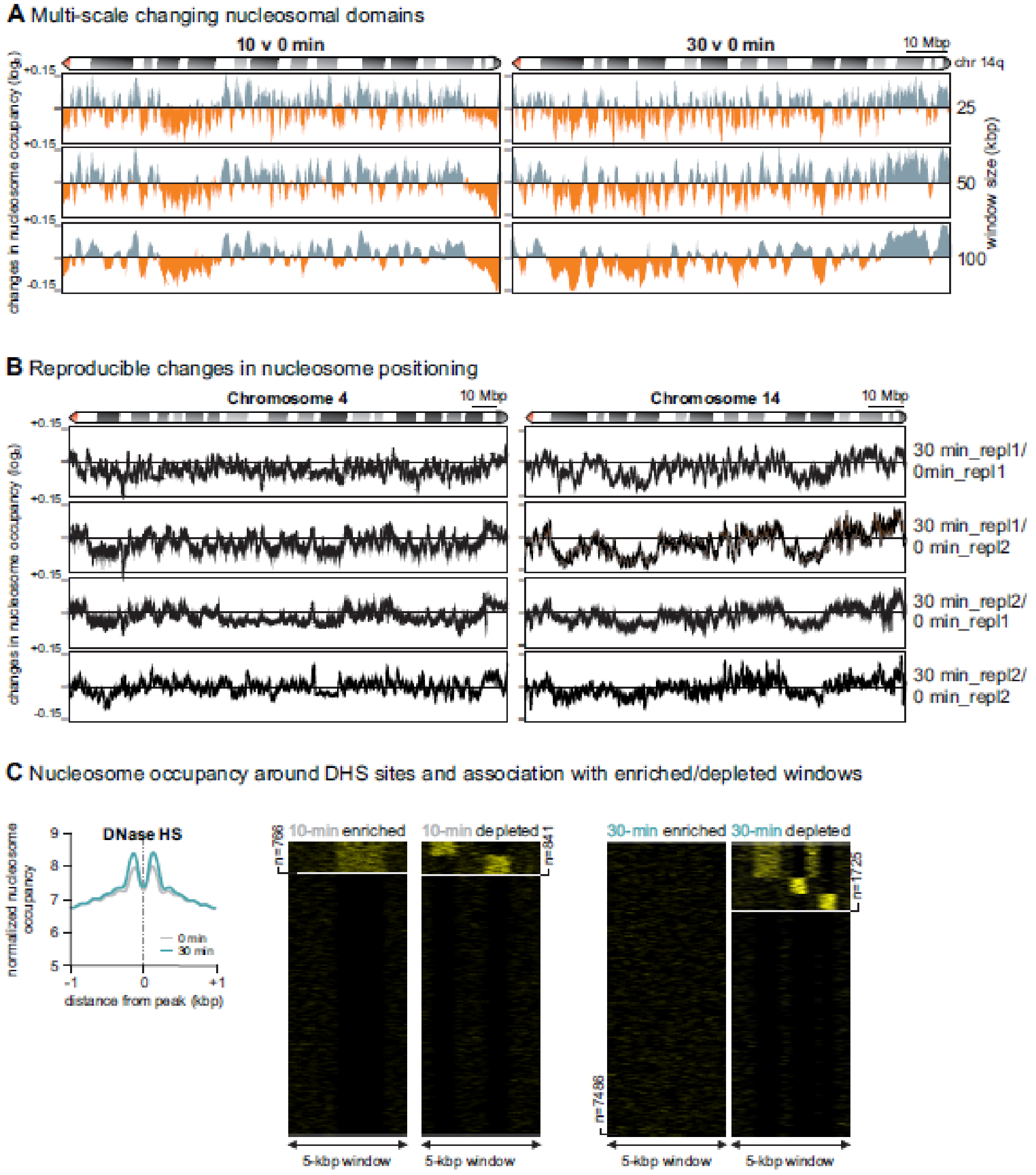

Additional file 8 | Multi-scale TNFα-responsive nucleosome domains. (**A**) Changes in nucleosome occupancy along chromosome 14 (*ideogram*) are shown at 10 or 30 min post-stimulation (log_2 fold changes in 25-, 50-, and 100-kbp non-overlapping windows, determined as in **Figure 2**). Profiles (enrichment: *grey*; depletion: *orange*) remain similar regardless of window size. (**B**) Reproducibility of observed changes in nucleosome occupancy (determined as in **Figure 2**). 0- and 30-min MNase-seq data from two biological replicates ("repl1" and "repl2"), used in pairwise combinations, yield similar profiles (Spearman's correlation >0.85) along chromosomes 4 and 14 (*ideograms*). (**C**) Left: A plot showing (normalized) nucleosome occupancy around DNase I-hypersensitive sites (DHS) at 0 and 30 min post-stimulation. *Right*: Using chromosome 14 as an example, DHS sites (ENCODE data, HUVECs) overlapping 10- or 30-min 5-kbp windows were recorded. Of 7,486 DHS sites interrogated, a marginal difference is observed between nucleosome-enriched (766 sites) and -depleted (841 sites) for the 10-min state, whereas for the 30-min one 1,725 DHS sites were found only in nucleosome-depleted windows.
