# Supplementary material for: TNFα signalling primes chromatin for NF-κB binding and induces rapid and widespread nucleosome repositioning
Source: Genome Biol. 2014 Dec 3;15(12):536. doi: 10.1186/s13059-014-0536-6 (PMC4268828; doi:10.1186/s13059-014-0536-6)
Supplement: Additional file 9: — Chromatin interaction analysis by paired-end tag sequencing supports 3C-seq data along two chromosomal loci. [file 13059_2014_536_MOESM9_ESM.pdf]

Additional File 9

3C-seq contacts around representative loci

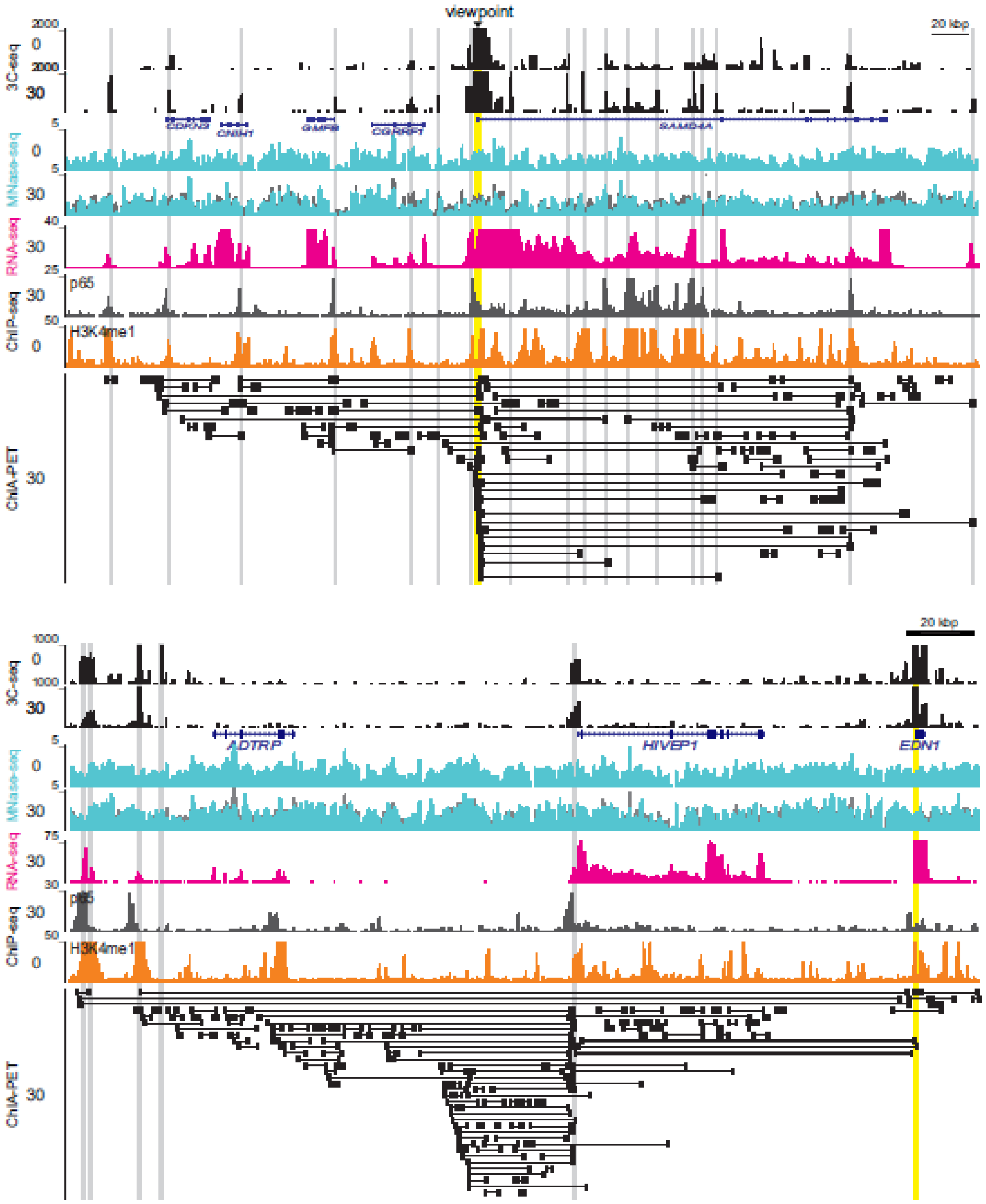

Additional file 9 | ChIA-PET supports 3C-seq data along two chromosomal loci. Browser views of TNFα-responsive *SAMD4A* and non-responsive *EDN1* loci (on chromosomes 14 and 6, respectively) showing tracks illustrating profiles obtained by 3C-seq (using *Apo*I; contacts detected from each TSS "viewpoint", *yellow highlight*), MNase-seq (0-min levels in *grey* underlie 30-min ones in *green* to facilitate comparison), RNA-seq (*magenta*), p65 (*black*) or H3K4me1 ChIP-seq (*orange*), and ChIA-PET (after an RNAP pull-down [18]; *black* lines connect contacting sequences). Strong contacts detected by 3C-seq (*grey highlights*) are supported by ChIA-PET, and often coincide with (or are adjacent to) transcribed regions binding p65 and bearing H3K4me1 marks; at these regions nucleosome repositioning is also observed.
